# The extent of grain yield and plant growth enhancement by plant growth-promoting broad-spectrum *Streptomyces* sp. in chickpea

**DOI:** 10.1186/s40064-015-0811-3

**Published:** 2015-01-23

**Authors:** Subramaniam Gopalakrishnan, Vadlamudi Srinivas, Gottumukkala Alekhya, Bandikinda Prakash, Himabindu Kudapa, Abhishek Rathore, Rajeev Kumar Varshney

**Affiliations:** International Crops Research Institute for the Semi-Arid Tropics (ICRISAT), Patancheru, 502 324 Telangana India

**Keywords:** Field evaluation, Plant growth-promotion (PGP), qRT-PCR analysis, Scanning electron microscopy, *Streptomyces* sp.

## Abstract

The physiological and molecular responses of five strains of *Streptomyces* sp. (CAI-17, CAI-68, CAI-78, KAI-26 and KAI-27), with their proven potential for charcoal rot disease control in sorghum and plant growth-promotion (PGP) in sorghum and rice, were studied to understand the mechanisms causing the beneficial effects. In this investigation, those five strains were evaluated for their PGP capabilities in chickpea in the 2012–13 and 2013–14 post-rainy seasons. All of the *Streptomyces* sp. strains exhibited enhanced nodule number, nodule weight, root weight and shoot weight at 30 days after sowing (DAS) and pod number, pod weight, leaf area, leaf weight and stem weight at 60 DAS in both seasons over the un-inoculated control. At crop maturity, the *Streptomyces* strains had enhanced stover yield, grain yield, total dry matter and seed number plant^−1^ in both seasons over the un-inoculated control. In the rhizosphere, the *Streptomyces* sp. also significantly enhanced microbial biomass carbon, dehydrogenase activity, total nitrogen, available phosphorous and organic carbon in both seasons over the un-inoculated control. Of the five strains of *Streptomyces* sp., CAI-17, CAI-68 and CAI-78 were superior to KAI-26 and KAI-27 in terms of their effects on root and shoot development, nodule formation and crop productivity. Scanning electron microscopy (SEM) micrographs had revealed the success in colonization of the chickpea roots by all five strains. Quantitative real-time PCR (qRT-PCR) analysis of selected PGP genes of actinomycetes revealed the selective up-regulation of indole-3-acetic acid (IAA)-related and siderophore-related genes by CAI-68 and of β-1,3-glucanase genes by KAI-26.

## Introduction

In recent years, the use of plant growth-promoting (PGP) bacteria has become one of the most attractive options for enhancing the sustainability of agricultural systems in many parts of the world due to their eco-friendliness, low production cost and reduced use of non-renewable resources. PGP bacteria promote plant growth through various mechanisms such as nitrogen fixation, the solubilization of phosphorus, the chelation of iron, the secretion of plant growth hormones and the inhibition of phytopathogens. Bacteria such as *Bacillus* sp., *Pseudomonas* sp. and *Streptomyces* sp. were found to play a major role both in mobilizing and acquiring nutrients and in controlling phytopathogens (Postma et al. 2003; Perner et al. 2006; Gopalakrishnan et al. 2011a, b, c; Jalilian et al. 2012). The presence of PGP bacteria in the rhizosphere has been shown to enhance shoot growth, root growth, root hair development, plant hormone concentrations, nitrogen fixation, the solubilization of minerals and the suppression of pathogens (Shaukat et al. 2006; Lucas et al. 2009; Richardson et al. 2009; Gopalakrishnan et al. 2012a, b). The PGP potential of *Streptomyces* sp. has been demonstrated on tomato, wheat, rice, bean and pea (Tokala et al. 2002; Nassar et al. 2003; El-Tarabily 2008; Sadeghi et al. 2012; Gopalakrishnan et al. 2013, 2014a). *Streptomyces* sp. are known to promote plant growth either by producing indole-3-acetic acid (IAA) and siderophores and/or by inhibiting soil-borne fungal pathogens (Aldesuquy et al. 1998; Trejo-Estrada et al. 1998; Tokala et al. 2002; Macagnan et al. 2008; Gopalakrishnan et al. 2011a, b, 2013; 2014a).

Previously, we have demonstrated the potential of a set of eight *Streptomyces* strains (CAI-21, CAI-26, MMA-32, CAI-17, CAI-68, CAI-78, KAI-26 and KAI-27) isolated from herbal vermicompost, for the bio-control of charcoal rot disease, caused by *Macrophomina phaseolina* (Tassi) Goid., in sorghum (Gopalakrishnan et al. 2011b) and for PGP in rice (Gopalakrishnan et al. 2012a, 2013). The objectives of this investigation were to further evaluate five of the eight *Streptomyces* strains (CAI-17, CAI-68, CAI-78, KAI-26 and KAI-27) for their PGP traits in chickpea under field conditions, to demonstrate gene expression profiles by qRT-PCR analysis, and to ensure colonizing ability in chickpea by scanning electron microscopy (SEM) analysis.

## Materials and methods

### *Streptomyces* strains

Five strains of *Streptomyces* spp., CAI-17 (GenBank accession number: JQ682619), CAI-68 (GenBank accession number: JQ682622), CAI-78 (GenBank accession number: JQ682623), KAI-26 (GenBank accession number: JQ682624) and KAI-27 (GenBank accession number: JQ682625), previously reported by us to have capacity for the bio-control of charcoal rot in sorghum (Gopalakrishnan et al. 2011b) and PGP in rice (Gopalakrishnan et al. 2013), were further studied in the present investigation.

### Evaluation of *Streptomyces* sp. for PGP potential on chickpea under field conditions

This experiment was carried out during the 2012–13 and 2013–14 post-rainy cropping seasons at ICRISAT, Patancheru (17°30′ N; 78°16′ E; altitude 549 m) in peninsular India. Soils at the experimental site are classified as Vertisols (fine montmorillonitic isohyperthermic typic pallustert) having 52% clay, 21% silt and 26% sand, with an alkaline pH of 7.7 − 8.5 and an organic carbon content of 0.4 − 0.6%. The soil depth of the field used was ≥ 1.2 m, and this soil retained approximately 200 mm of plant-available water in a 120-cm (maximum rooting depth by chickpea) soil profile. The mineral content of the top 15 cm of rhizosphere soil includes 24.7 mg kg^−1^ soil of available nitrogen, 8.6 mg kg^−1^ soil of available phosphorous and 298 mg kg^−1^ soil of available potassium. The field was kept fallow except for this post-rainy season crop. The fields were prepared into broad beds and furrows with beds 1.2 m wide flanked by 0.3-m furrows in both seasons. Surface application and incorporation of 18 kg N ha^−1^ and 20 kg P ha^−1^ as di-ammonium phosphate (DAP) were performed in both seasons. The experiment was laid out in a randomized complete block design (RCBD) with three replicates and plot sizes of 4 m × 3 ridges (rows).

The five strains of *Streptomyces* spp. were cultured individually on a starch casein broth at 28°C for five days. Seeds of chickpea variety ICCV 2 (with a grain yield potential of 1.1 to 1.2 t ha^−1^) were treated with a *Streptomyces* sp. (containing 10^8^ CFU ml^−1^) for 50 min and sown by hand on 11 October 2012 in the first year and 1 November 2013 in the second year in rows 30 cm apart at a depth of 4–5 cm to achieve an estimated plant stand density of at least 26 plants m^−2^. *Streptomyces* sp. (1000 ml; 10^8^ CFU mL^−1^) was applied once every 15 days on the soil close to the plant until the flowering stage. Control plots were maintained without the application of *Streptomyces* strains. The plots were irrigated on the 21^st^ and 49^th^ days after sowing. The crop was kept weed-free by manual weeding. No serious insect pest or phytopathogen attacks were observed during the cropping period. The crop was harvested manually on 18 January 2013 in the first year and 6 Feb 2014 in the second year. In both the 2012–13 and 2013–14 seasons, the nodule number (plant^−1^), nodule weight (mg plant^−1^), root weight (mg plant^−1^) and shoot weight (g plant^−1^) were recorded at 30 days after sowing (DAS), and at 60 DAS, the plant height (cm), pod number (plant^−1^), pod weight (g plant^−1^), leaf area (cm^−2^ plant^−1^) and stem weight (g plant^−1^) were recorded. At both harvests, stover yield (t ha^−1^), grain yield (t ha^−1^), total dry matter (t ha^−1^), 1000-seed weight (g), pod weight (g plant^−1^), seed number (plant^−1^) and seed weight (g plant^−1^) were recorded. Soil samples were collected from the top 15 cm of the soil profile at the harvest stage and analysed for soil chemistry (total nitrogen [ppm], available phosphorous [ppm] and % organic carbon as per the protocols of Novozamsky et al. 1983, Olsen and Sommers 1982 and Nelson and Sommers 1982, respectively) and microbial biomass carbon (μg g^−1^ soil) by the fumigation method and dehydrogenase activity (μg TPF g^−1^ soil 24 h^−1^) by the triphenyl formazan production method as per the protocols of Anderson and Domsch 1989 and Casida 1977, respectively.

### Colonization of *Streptomyces* sp.

The roots of chickpea were examined for colonization by *Streptomyces* sp. by SEM (Bozzola and Russell 1998). For this procedure, the seeds of chickpea variety ICCV 2 were surface-sterilized first with 2.5% sodium hypochlorite solution (for five minutes), followed by 70% ethanol (for five minutes), and then rinsed with sterilized water (eight times) before being allowed to sprout in a Petri plate overnight. The sprouted seeds were transferred into test *Streptomyces* strains (CAI-17, CAI-68, CAI-78, KAI-26 and KAI-27; grown in Bennett’s broth separately) for an hour before being sown in pots containing sterilized coarse sand (six seeds/8″ plastic pot). The soil was drenched with a booster dose of *Streptomyces* strains (5 ml per seedling; 10^8^ CFU ml^−1^) one week after sowing in sand. The pots were incubated for two weeks in a greenhouse at a temperature of 24 ± 2°C. At the end of the incubation period, chickpea seedlings were removed from the sand pots and the roots were washed in phosphate buffer (0.1 M; pH 7.2). The tip of the roots were cut into pieces 5 mm long and fixed in 2.5% glutaraldehyde in phosphate buffer for 24 h at 4°C. At the end of incubation, the samples were washed with phosphate buffer, postfixed in 2% osmium tetroxide for 4 h and dehydrated using a graded series of ethanol. The dehydrated samples were dried with critical-point liquid carbon dioxide as a transition fluid. The dried materials were adhered onto aluminium specimen mounts with double-stick adhesive tape. The samples were later coated with gold-palladium in an automated sputter coater (JEOL JFC-1600) and examined with a scanning electron microscope (JOEL-JSM 5600) as per the standardized protocols at RUSKA lab, College of Veterinary Science, Rajendranagar, Hyderabad, India. Observations of the presence of *Streptomyces* spores on root surfaces were recorded.

### Gene expression profile

#### RNA extraction

The five selected *Streptomyces* sp. (CAI-17, CAI-68, CAI-78, KAI-26 and KAI-27) were grown in Bennett’s broth at 28°C for 72 h. Total RNA was extracted from all of the *Streptomyces* strains using the “NucleoSpin® RNA Plant” kit (Macherey-Nagel). The quality and quantity of RNA was estimated by a Nanodrop (Thermo Scientific) and RNA integrity was determined using a 2100 Bioanalyzer (Agilent).

#### Quantitative real time- PCR (qRT-PCR)

*qRT-PCR* was performed using the Applied Biosystems 7500 Real-Time PCR System with the SYBR green chemistry (Applied Biosystems, USA) as per the manufacturer’s instructions. Well-characterized genes Spaepen et al. 2007 relating to IAA production (F: GTCACCGGGATCTTCTTCAAC; R: GATGTCGGTGTTCTTGTCCAG) and siderophore (F: ATCCTCAACACCCTGGTCTG; R: TCCTTGTACTGGTACGGGACTT) were collected from the UniprotKB database (http://www.uniprot.org/uniprot/) as described by Gopalakrishnan et al. (2014a). Similarly, genes for β-1,3-glucanase (F: CCGAACACCACCTACTCCAC; R: CCAGGTTGAGGATCAGGAAG) were also selected for the study. Gene-specific primers for real-time PCR were designed using Primer 3 software (Rosen and Skaletsky 2000). RNA polymerase principal sigma factor *HrdB* (SCO5820) (F: GGTCGAGGTCATCAACAAGC; R: CTCGATGAGGTCACCGAACT) was used as the endogenous control. qRT-PCR reactions were performed as described earlier (Gopalakrishnan et al. 2014a). The data obtained were analysed using the mean of the CT values of the three biological replicates that were normalized to the mean CT values of the endogenous gene. The expression ratios were calculated using the 2^_∆∆Ct^ method. Relative transcription levels are presented graphically.

### Statistical analysis

Analysis of variance) was performed by the GLM (General Linear Model) procedure of SAS software package (SAS Institute Inc 2013). Isolate were tested for significance and their means with control were compared by using Dunnett’s *t*-test.

## Results

As all of the strains of *Streptomyces* spp. (CAI-17, CAI-68, CAI-78, KAI-26 and KAI-27) enhanced different plant component performances, the means of all the five strains are presented. The *Streptomyces*-treated plots, by a mean of the five strains, enhanced agronomic performance in terms of nodule numbers (up to 61%), nodule weight (up to 44%) and shoot weight (up to 28%) at 30 DAS and plant height (up to 5%), pod numbers (up to 79%), pod weight (up to 89%), leaf area (up to 29%), leaf weight (up to 42%) and stem weight (up to 6%) at 60 DAS in both seasons (2012–13 and 2013–2014) over the un-inoculated control plots (Tables 1 and 2). At chickpea crop maturity, the *Streptomyces*-treated plots also enhanced stover yield (up to 41%), grain yield (up to 16%), total dry matter (up to 26%), pod weight (up to 7%), seed number (up to 21%) and seed weight (up to 11%) in both seasons over the un-inoculated control plots (Table 3). At crop maturity, in the top 15-cm of rhizosphere soil, the *Streptomyces* sp. treatments enhanced the microbial biomass carbon (up to 59%) and dehydrogenase activity (up to 21%), as well as soil mineral nutrient contents, including total N (up to 8%), available P (up to 45%) and % organic carbon (up to 7%), in both seasons over the un-inoculated control plots (Tables 4 and 5).

**Table 1:** **Response of various plant components of chickpea to the application of different strains of** ***Streptomyces*** **sp. at 30 days after sowing in a Vertisol during 2012–13 and 2013–14 post-rainy seasons**

	**Year 2012–13**				**Year 2013–14**			
**Isolate**	**Nodule number (plant** ^**−1**^ **)**	**Nodule weight (mg plant** ^**−1**^ **)**	**Root weight (mg plant** ^**−1**^ **)**	**Shoot weight (g plant** ^**−1**^ **)**	**Nodule number (plant** ^**−1**^ **)**	**Nodule weight (mg plant** ^**−1**^ **)**	**Root weight (mg plant** ^**−1**^ **)**	**Shoot weight (g plant** ^**−1**^ **)**
CAI-17	20^*^	36	186^*^	2.11^*^	51	228	192	1.76
CAI-68	19^*^	37	173	1.76^*^	60^*^	240^*^	205^*^	1.91^*^
CAI-78	21^*^	71^*^	184	1.81^*^	59^*^	242^*^	207^*^	1.87
KAI-26	20^*^	29	172	1.39	50	228	183	1.75
KAI-27	19^*^	36	172	1.55	50	224	186	1.74
Control	12	29	171	1.35	49	221	168	1.72
Mean	19	40	176	1.66	53	231	190	1.79
SEM±	1.04^***^	4.1^***^	3.3^*^	0.06^***^	0.8^***^	4.2^*^	5.75^***^	0.04^*^
CV%	10	18	3	6	3	3	5	4

SEM = Standard Error of Mean; CV = Coefficients of variation; * = significant at p < 0.05, *** = significant at p < 0.001; According to Dunnett’s *t*-test, Isolate mean values are compared with control at p < 0.05.

**Table 2:** **Response of various plant components of chickpea to the application of different strains of** ***Streptomyces*** **sp. at 60 days after sowing in a Vertisol during 2012–13 and 2013–14 post-rainy seasons**

	**Year 2012–13**						**Year 2013–14**					
**Isolate**	**Plant height (cm)**	**Pod number (plant** ^**−1**^ **)**	**Pod weight (g plant** ^**−1**^ **)**	**Leaf area (cm** ^**−2**^ ** plant** ^**−1**^ **)**	**Leaf weight (g plant** ^**−1**^ **)**	**Stem weight (g plant** ^**−1**^ **)**	**Plant height (cm)**	**Pod number (plant** ^**−1**^ **)**	**Pod weight (g plant** ^**−1**^ **)**	**Leaf area (cm** ^**−2**^ **plant** ^**−1**^ **)**	**Leaf weight (g plant** ^**−1**^ **)**	**Stem weight (g plant** ^**−1**^ **)**
CAI-17	54^*^	65^*^	3.26^*^	1042^*^	7.25^*^	6.55^*^	49^*^	58	4.18	781^*^	4.45	4.17
CAI-68	57^*^	72^*^	4.47^*^	868^*^	5.30^*^	4.87^*^	48	66^*^	4.32	667	4.05	4.75^*^
CAI-78	51	91^*^	3.41^*^	770^*^	6.39^*^	4.92^*^	50^*^	87^*^	5.58^*^	878^*^	4.67	4.43
KAI-26	51	72^*^	3.52^*^	906^*^	6.38^*^	5.18^*^	49^*^	58	4.18	832^*^	4.54	4.17
KAI-27	48	83^*^	4.50^*^	739	5.56^*^	4.75^*^	47	56	4.49^*^	633	4.00	4.18
Control	50	43	2.04	670	4.36	3.36	47	57	4.14	626	3.71	4.11
Mean	52	71	3.53	833	5.87	4.94	48	64	4.48	736	4.24	4.30
SE±	0.4^***^	1.9^***^	0.151^***^	31.0^***^	0.212^***^	0.071^***^	0.6^*^	3.3^***^	0.079^***^	15.1^***^	0.199^*^	0.096^**^
CV%	1	5	7	6	6	3	2	9	3	4	8	4

SEM = Standard Error of Mean; CV = Coefficients of variation; * = significant at p < 0.05, ** = significant at p < 0.01, *** = significant at p < 0.001; According to Dunnett’s *t*-test, isolate mean values re compared with control at p < 0.05.

**Table 3:** **Response of grain yield and its components to the application of different strains of** ***Streptomyces*** **sp. to chickpea in a Vertisol during 2012–13 and 2013–14 post-rainy seasons**

	**Year 2012–13**							**Year 2013–14**						
**Isolate**	**Stover yield (t ha** ^**−1**^ **)**	**Grain yield (t ha** ^**−1**^ **)**	**TDM (t ha** ^**−1**^ **)**	**1000-seed weight (g)**	**Pod weight (g plant** ^**−1**^ **)**	**Seed number (plant** ^**−1**^ **)**	**Seed weight (g plant** ^**−1**^ **)**	**Stover yield (t ha** ^**−1**^ **)**	**Grain yield (t ha** ^**−1**^ **)**	**TDM (t ha** ^**−1**^ **)**	**1000-seed weight (g)**	**Pod weight (g plant** ^**−1**^ **)**	**Seed number (plant** ^**−1**^ **)**	**Seed weight (g plant** ^**−1**^ **)**
CAI-17	1.42^*^	2.01^*^	3.43^*^	220	22.2^*^	72^*^	16.7	1.75	1.70^*^	3.45	196	18.0^*^	72	15.0*
CAI-68	1.31^*^	1.89^*^	3.20^*^	221	18.2	72^*^	15.4	2.03	1.70^*^	3.72^*^	198^*^	17.3	70	15.3*
CAI-78	1.91^*^	2.16^*^	4.08^*^	227^*^	18.2	55	14.1	2.19^*^	1.84^*^	4.03^*^	200^*^	21.0^*^	82	15.9^*^
KAI-26	1.33^*^	1.96^*^	3.28^*^	231^*^	19.8^*^	63^*^	14.6	1.69	1.76^*^	3.45	196	17.4	65^*^	14.3
KAI-27	1.81^*^	1.81^*^	3.62^*^	222	17.2	63^*^	15.4	1.82	1.68^*^	3.51	196	17.5	67	13.6
Control	1.11	1.69	2.80	220	18.0	54	13.8	1.68	1.55	3.23	195	17.0	66	13.3
Mean	1.48	1.92	3.40	223	18.9	63	15.0	1.86	1.71	3.57	197	18.0	70	14.6
SEM±	0.020^***^	0.022^***^	0.034^***^	1.4^***^	0.24^***^	0.5^***^	0.42^**^	0.093^*^	0.019^***^	0.098^**^	0.7^**^	0.23^***^	3.3^*^	0.30^***^
CV%	2	2	2	1	2	2	5	9	2	5	1	2	8	4

TDM = total dry matter; SEM = Standard Error of Mean; CV% = Coefficients of variation; * = significant at p < 0.05, ** = significant at p < 0.01, *** = significant at p < 0.001; According to Dunnett’s *t*-test, isolate mean valus are compared with control at p < 0.05.

**Table 4:** **Response of microbial biomass carbon and dehydrogenase activity in the chickpea rhizosphere to the application of different strains of** ***Streptomyces*** **spp. in a Vertisol during 2012–13 and 2013–14 post-rainy seasons**

	**Year 2012–13**		**Year 2013–14**	
**Isolate**	**Microbial biomass C (μg g** ^**−1**^ ** soil)**	**Dehydrogenase activity (μg TPF g** ^**−1**^ ** soil 24 h** ^**−1**^ **)**	**Microbial biomass C (μg g** ^**−1**^ ** soil)**	**Dehydrogenase activity (μg TPF g** ^**−1**^ ** soil 24 h** ^**−1**^ **)**
CAI-17	1221^*^	52.7	974^*^	68.6^*^
CAI-68	1295^*^	56.8	1000^*^	71.5^*^
CAI-78	1424^*^	63.6^*^	908^*^	67.8
KAI-26	893^*^	53.0	842^*^	68.8^*^
KAI-27	1132^*^	56.8	444	59.0
Control	752	51.9	550	55.7
Mean	1119	55.8	786	65.2
SEM (±)	6.7^***^	1.74^**^	47.1^**^	2.41^*^
CV%	1	4	9	5

SEM = Standard Error of Mean; CV = Coefficients of variation; * = significant at p < 0.05, ** = significant at p < 0.01, *** = significant at p < 0.001; According to Dunnett’s *t*-test, isolate mean values are compared with control at p < 0.05.

**Table 5:** **Response of rhizosphere soil chemical properties of chickpea at its maturity to the application of different strains of** ***Streptomyces*** **sp. in a Vertisol during the 2012–13 and 2013–14 post-rainy seasons**

	**Year 2012–13**			**Year 2013–14**		
**Isolate**	**Total N (ppm)**	**Available P (ppm)**	**Organic carbon (%)**	**Total N (ppm)**	**Available P (ppm)**	**Organic carbon (%)**
CAI-17	656	11.2^*^	0.51^*^	737	11.1^*^	0.56
CAI-68	703^*^	10.4	0.49^*^	744	10.2	0.54
CAI-78	714^*^	12.2^*^	0.56^*^	815^*^	11.3^*^	0.67^*^
KAI-26	666	10.8^*^	0.45	724	10.2	0.57
KAI-27	668	10.8^*^	0.47	720	6.9	0.58
Control	632	10.1	0.47	735	6.9	0.55
Mean	673	10.9	0.49	746	9.4	0.58
SEM±	13.6^*^	0.13^***^	0.004^***^	15.2^*^	0.67^**^	0.016^*^
CV%	3	2	1	3	10	4

SEM = Standard Error of Mean; CV = Coefficients of variation; * = significant at p < 0.05, ** = significant at p < 0.01, *** = significant at p < 0.001; According to Dunnett’s *t*-test, isolate mean values are compared with control at p < 0.05.

**Figure 1:**
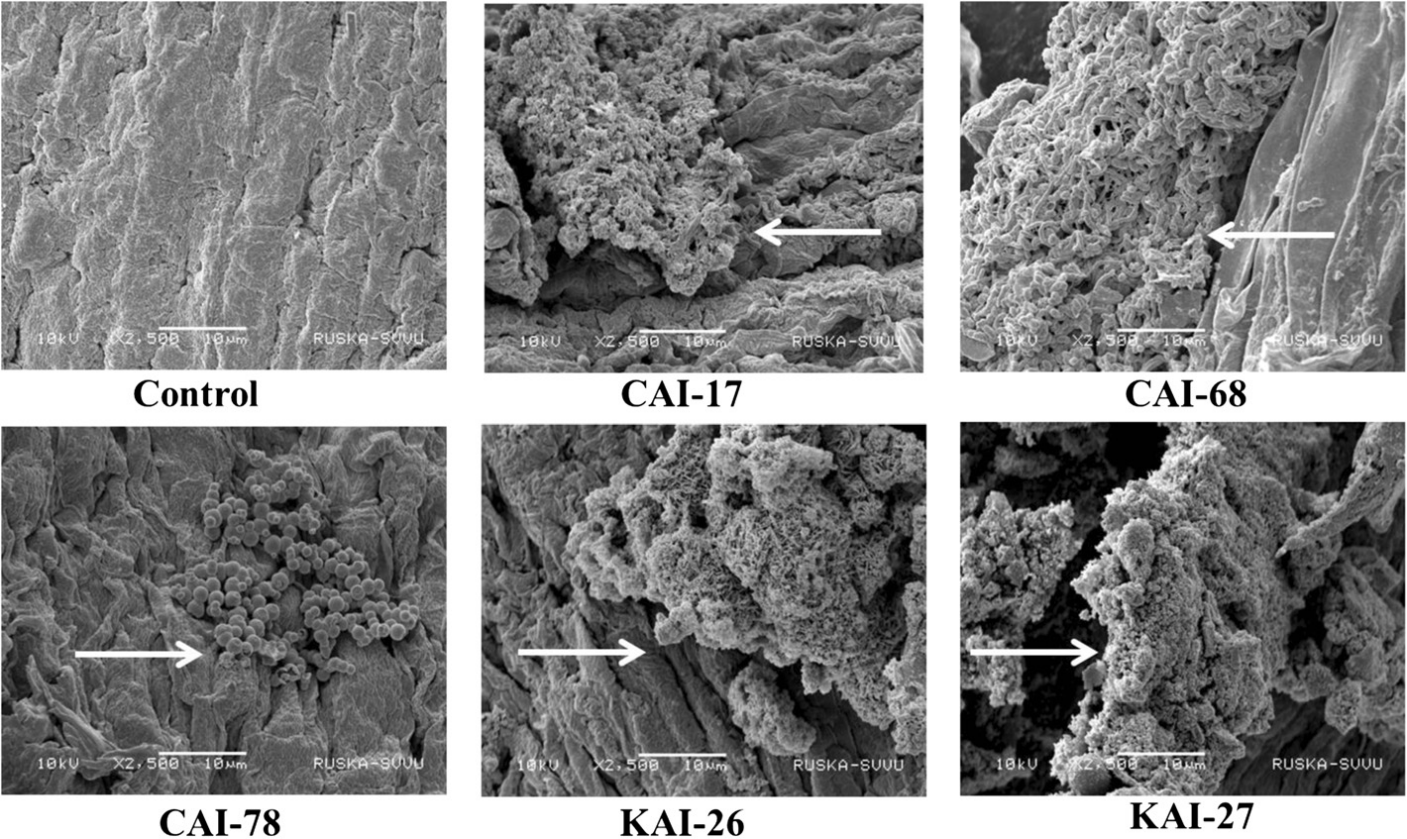
**Scanning electron microscopy photographs of the five** ***Streptomyces*** **sp. showing colonization on the roots of chickpea.** Note: Arrows indicate chickpea roots colonized by PGP Streptomyces strains CAI-17, CAI-68, CAI-78, KAI-26, and KAI-27.

SEM analysis of chickpea roots revealed a remarkable degree of colonization by *Streptomyces* sp. Roots from inoculated plants exhibited significant surface colonization by *Streptomyces* sp., while those from un-inoculated plants did not. The sporulation of *Streptomyces* sp. on the surface cell layer of chickpea roots was clearly evident for all five strains. The hyphae of *Streptomyces* sp. were also found to penetrate the surface cell layer of chickpea roots (Figure 1).

**Figure 2:**
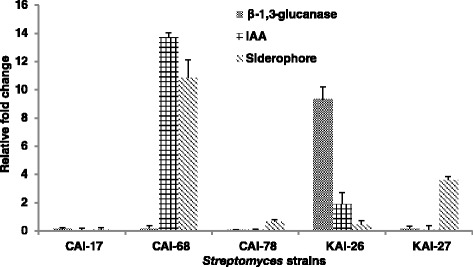
**Expression profiling of PGP genes of** ***Streptomyces*** **sp. through quantitative real-time PCR assays.**

Gene expression profiles of plant-growth-promoting genes, IAA, siderophore and β-1,3-glucanase revealed overall up-regulation in the *Streptomyces* strains studied. The gene IAA showed higher up-regulation in the strain CAI-68 (13.7-fold), while no significant variation in the expression pattern was observed in the other four strains. The siderophore gene was highly up-regulated in strain CAI-68 (10.8-fold), followed by KAI-27 (3.6), and β-1,3-glucanase expression was up-regulated in KAI-26 (9.3-fold) (Figure 2).

## Discussion

The five strains of *Streptomyces* spp. (CAI-17, CAI-68, CAI-78, KAI-26 and KAI-27), which are known for biocontrol of charcoal rot in sorghum and PGP in sorghum (Gopalakrishnan et al. 2011b) and rice (Gopalakrishnan et al. 2013), were evaluated for their PGP effects in chickpea under field conditions. In the present study, nodules were observed in the roots of chickpea on both the seasons (2012–13 and 2013–2014) though rhizobia were neither inoculated on seed nor applied on soil indicating the presence of native rhizobia in the soil. Rhizobia were always noted in the Vertisols of ICRISAT (Gopalakrishnan et al. 2014b). The increase in the number of nodules per plant in 2013–14 over 2012–13 could be due to the build-up of native rhizobia specific to chickpea during the cropping season of 2012–13 and hence subsequently observed in the following season, 2013–14. Of the five *Streptomyces* sp. applied, CAI-78 significantly enhanced all of the agronomic characteristics and yield components the most, including the nodule numbers and nodule weight. The enhancement effects of the other strains were, in descending order, CAI-68, CAI-17, KAI-26 and KAI-27.

The highest magnitude of enhanced soil biological activities and mineral nutrient properties was found with CAI-78 treatment, followed by CAI-68, CAI-17, KAI-26 and KAI-27. The enzyme dehydrogenase is regarded as an indicator of total life in the soil and a strong indicator of biological activity. Similarly, increased microbial biomass C and dehydrogenase activity have been reported in fields upon the use of biological options for soil fertilization and insect-pests control (Carpenter-Boggs et al. 2000; Sharma and Singh 2004). Superior soil nutrient status (N, P, K and % organic carbon) has been reported on organic farms compared to soils treated with chemical pesticides and fertilizers (Sharma and Singh 2004; Singh et al. 2004). In the present study, the enhancement of soil biological and mineral nutrient properties with *Streptomyces* treated plots over un-inoculated control could be due to the presence of more microbial activity in the rhizosphere which leads to beneficial functions for crops such as plant growth-promotion, nitrogen fixation, phosphate solubilization and protection against pathogens.

The PGP influence of *Streptomyces* sp. had previously been reported in various agriculturally important crops (Tokala et al. 2002; Nassar et al. 2003; El-Tarabily 2008; Sadeghi et al. 2012; Gopalakrishnan et al. 2014a). PGP agents having a broad spectrum of activities offer effective and novel strategies not only for the improvement of crop growth and yield but also for the control of insect pests and pathogens that affect crop plants. In addition to suppressing plant pathogens and insect pests by secreting secondary metabolites, such as antibiotics, some PGP agents also elicit induced systemic resistance against a broad range of insects and pathogens (Jetiyanon and Kloepper 2002; Ryu et al. 2007). Although the *Streptomyces* spp. used in this study excrete antimicrobial compounds, antagonism tests by the poison food technique indicate that none of the strains inhibit the growth of nodulating bacteria *Rhizobium leguminosarum* on yeast extract mannitol agar media (data not shown). Thus, the *Streptomyces* spp., appears to be compatible with *Rhizobium*.

Colonization of chickpea roots by *Streptomyces* spp., at the right time and place is essential for enhanced PGP activity. Successful host-microbe interaction is essential, which depends on the presence of a sufficient population of bacteria, as well as the rhizosphere competence, root-colonizing ability and PGP capability of the bacteria (Lugtenberg and Dekkers 1999). The data for grain and stover yield, root mass and other agronomical traits, the chemical and biological activities of the rhizosphere soil along with the SEM micrograph clearly establish that the PGP effects of the *Streptomyces* spp., had been caused by successful colonization of the inoculated chickpea roots.

The mechanism by which the five *Streptomyces* sp. could consistently enhance agronomical and yield traits on sorghum, rice (from our previous study) and chickpea can be attributed to their enzymatic activities, such as the ability to produce siderophores and indole acetic acid and β-1,3-glucanase (Gopalakrishnan et al. 2011b; 2013). Siderophores are known to form stable organic complexes with heavy metals such as Cu, Cd, In, U, Np, Al, Pb, Zn and Ga and increase the soluble metal concentration (Rajkumar et al. 2010) in the soil system, thus alleviating the various heavy metal stresses imposed on plants. Siderophores also act as solubilizing agents of iron from minerals when iron nutrition becomes a limitation (Indiragandhi et al. 2008). IAA-producing microorganisms are known to stimulate seed germination, initiate adventitious and lateral root formation and increase root length and surface area, thereby providing the host plant greater access to soil nutrients and water (Ahemad and Kibret 2014). The cell wall of plant pathogens is composed of layers of β-1,3-glucan (as in the case of *Fusarium oxysporum*, the causal agent of wilt disease in many crops) and lysis of these layers by β-1,3-glucanase-producing microorganisms leads to the leakage of cell contents and the collapse of the pathogenic fungi (Singh et al. 1999).

Our previous results documented the production of IAA (0.22 – 0.95%), β-1,3 glucanase (0.2 – 2.92 units) and siderophore (qualitative; 1–3 rating scale) by all of these *Streptomyces* strains (Gopalakrishnan et al. 2011b; Gopalakrishnan et al. 2013). Further characterization on gene expression profiles of these PGP genes revealed overall up-regulation in all the *Streptomyces* strains. However, no significant change in expression profiles of all three genes, IAA, siderophore and β-1,3-glucanase, was observed in isolates CAI-17 and CAI-78. The relative expression profiles of these genes in CAI-17 and CAI-78 do not correlate with the PGP capabilities observed under field conditions. This may be due to analysis of the gene expression profiles in only one biological replication of the strains. The exact role of these genes is to be precisely elucidated with more biological replications. However, validation of the IAA, siderophore and β-1,3-glucanase genes confirmed the insights gained from *in vitro* PGP attributes of the studied *Streptomyces* strains. Hence, it is concluded that the *Streptomyces* strains studied in this investigation contains multiple PGP traits and can therefore be exploited not only for PGP but also for biological control of plant pathogens.

The five *Streptomyces* spp. used in this study are apparently well adapted not only to the sorghum and rice rhizospheres, as reported earlier, but also to the chickpea rhizosphere, as demonstrated in the current investigation. Additionally, the five *Streptomyces* spp. contain a broad range of PGP abilities and demonstrate multiple beneficial actions. Therefore, the five *Streptomyces* spp. used in this study are potential candidates for the discovery of novel secondary metabolites and their usefulness in host plant resistance against a range of pathogens and insect pests. These *Streptomyces* sp. strains can be useful in furthering the use of eco-friendly bio-pesticides and bio-fertilizers in integrated pest, disease and nutrition management programs. However, there is a need to determine the effectiveness of these *Streptomyces* strains under multi-location trials and to understand the nature of their interaction with other native soil micro-flora and micro-fauna with host plants and the environment.
